# Male-to-Female Gender-Affirming Surgery: 20-Year Review of Technique and Surgical Results

**DOI:** 10.3389/fsurg.2021.639430

**Published:** 2021-05-05

**Authors:** Gabriel Veber Moisés da Silva, Maria Inês Rodrigues Lobato, Dhiordan Cardoso Silva, Karine Schwarz, Anna Martha Vaitses Fontanari, Angelo Brandelli Costa, Patric Machado Tavares, Antonio Rebello Horta Gorgen, Renan Desimon Cabral, Tiago Elias Rosito

**Affiliations:** ^1^Serviço de Urologia, Hospital de Clínicas de Porto Alegre, Porto Alegre, Brazil; ^2^Serviço de Psiquiatria, Hospital de Clínicas de Porto Alegre, Porto Alegre, Brazil; ^3^Serviço de Psiquiatria, Pontifical Catholic University of Rio Grande do Sul, Porto Alegre, Brazil

**Keywords:** transsexualism, gender dysphoria, gender-affirming genital surgery, penile inversion vaginoplasty, surgical outcome

## Abstract

**Purpose:** Gender dysphoria (GD) is an incompatibility between biological sex and personal gender identity; individuals harbor an unalterable conviction that they were born in the wrong body, which causes personal suffering. In this context, surgery is imperative to achieve a successful gender transition and plays a key role in alleviating the associated psychological discomfort. In the current study, a retrospective cohort, we report the 20-years outcomes of the gender-affirming surgery performed at a single Brazilian university center, examining demographic data, intra and postoperative complications. During this period, 214 patients underwent penile inversion vaginoplasty.

**Results:** Results demonstrate that the average age at the time of surgery was 32.2 years (range, 18–61 years); the average of operative time was 3.3 h (range 2–5 h); the average duration of hormone therapy before surgery was 12 years (range 1–39). The most commons minor postoperative complications were granulation tissue (20.5 percent) and introital stricture of the neovagina (15.4 percent) and the major complications included urethral meatus stenosis (20.5 percent) and hematoma/excessive bleeding (8.9 percent). A total of 36 patients (16.8 percent) underwent some form of reoperation. One hundred eighty-one (85 percent) patients in our series were able to have regular sexual intercourse, and no individual regretted having undergone GAS.

**Conclusions:** Findings confirm that it is a safety procedure, with a low incidence of serious complications. Otherwise, in our series, there were a high level of functionality of the neovagina, as well as subjective personal satisfaction.

## Introduction

Transsexualism (ICD-10) or Gender Dysphoria (GD) (DSM-5) is characterized by intense and persistent cross-gender identification which influences several aspects of behavior (1). The terms describe a situation where an individual's gender identity differs from external sexual anatomy at birth (1). Gender identity-affirming care, for those who desire, can include hormone therapy and affirming surgeries, as well as other procedures such as hair removal or speech therapy (1).

Since 1998, the Gender Identity Program (PROTIG) of the Hospital de Clínicas de Porto Alegre (HCPA), Universidade Federal do Rio Grande do Sul, Brazil has provided public assistance to transsexual people, is the first one in Brazil and one of the pioneers in South America. Our program offers psychosocial support, health care, and guidance to families, and refers individuals for gender-affirming surgery (GAS) when indicated. To be eligible for this surgery, transsexual individuals must have been adherent to multidisciplinary follow-up for at least 2 years, have a minimum age of 21 years (required for surgical procedures of this nature), have a positive psychiatric or psychological report, and have a diagnosis of GD.

Gender-affirming surgery (GAS) is increasingly recognized as a therapeutic intervention and a medical necessity, with growing societal acceptance (2). At our institution, we perform the classic penile inversion vaginoplasty (PIV), with an inverted penis skin flap used as the lining for the neovagina. Studies have demonstrated that GAS for the management of GD can promote improvements in mental health and social relationships for these patients (2–5). It is therefore imperative to understand and establish best practice techniques for this patient population (2). Although there are several studies reporting the safety and efficacy of gender-affirming surgery by penile inversion vaginoplasty, we present the largest South-American cohort to date, examining demographic data, intra and postoperative complications.

## Patients and Methods

### Subjects and Study Setup

This is a retrospective cohort study of Brazilian transgender women who underwent penile inversion vaginoplasty between January of 2000 and March of 2020 at the Hospital de Clínicas de Porto Alegre, Porto Alegre, Brazil. The study was approved by our institutional medical and research ethics committee.

At our institution, gender-affirming surgery is indicated for transgender women who are under assistance by our program for transsexual individuals. All transsexual women included in this study had at least 2 years of experience as a woman and met WPATH standards for GAS (1). Patients were submitted to biweekly group meetings and monthly individual therapy.

Between January of 2000 and March of 2020, a total of 214 patients underwent penile inversion vaginoplasty. The surgical procedures were performed by two separate staff members, mostly assisted by residents. A retrospective chart review was conducted recording patient demographics, intraoperative and postoperative complications, reoperations, and secondary surgical procedures. Informed consent was obtained from all individual participants included in the study.

### Hormonal Therapy

The goal of feminizing hormone therapy is the development of female secondary sex characteristics, and suppression/minimization of male secondary sex characteristics.

Our general therapy approach is to combine an estrogen with an androgen blocker. The usual estrogen is the oral preparation of estradiol (17-beta estradiol), starting at a dose of 2 mg/day until the maximum dosage of 8 mg/day. The preferred androgen blocker is spironolactone at a dose of 200 mg twice a day.

### Operative Technique

**Figure 1:**
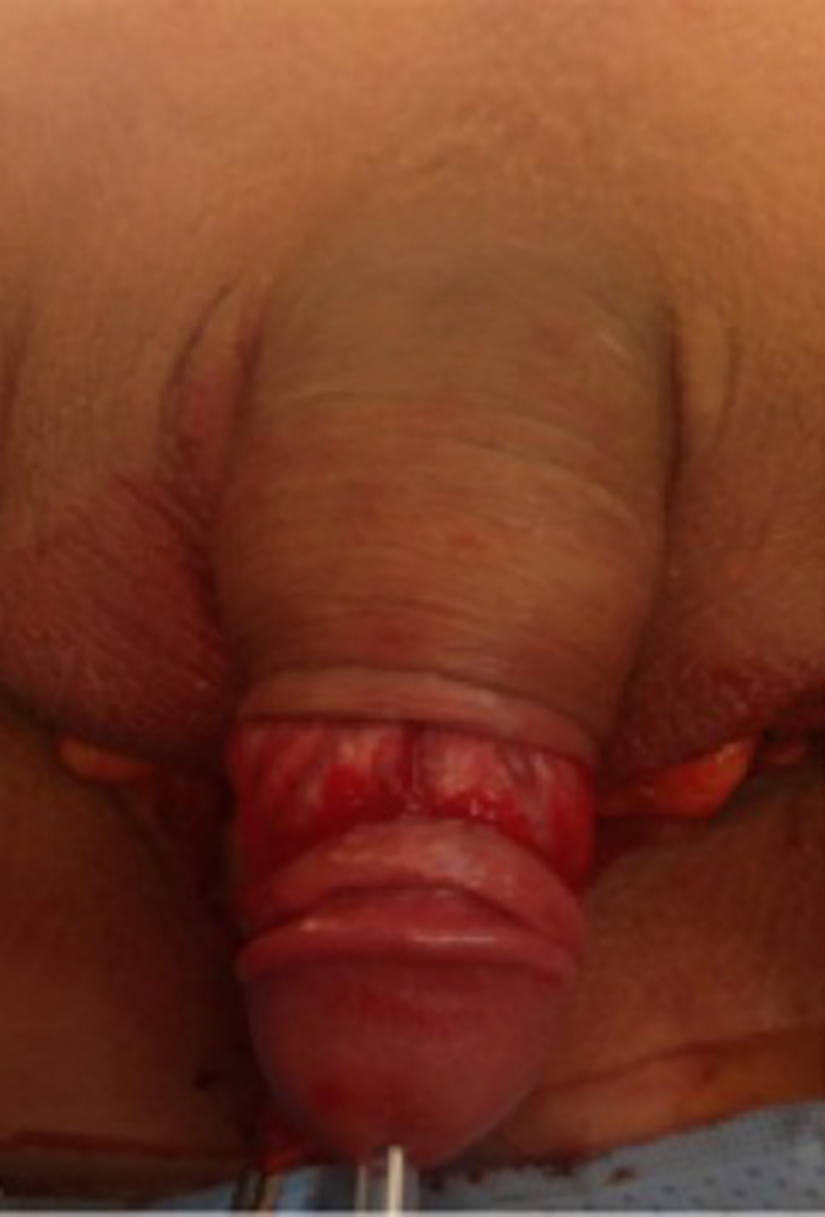
The initial circumferential subcoronal incision.

**Figure 2:**
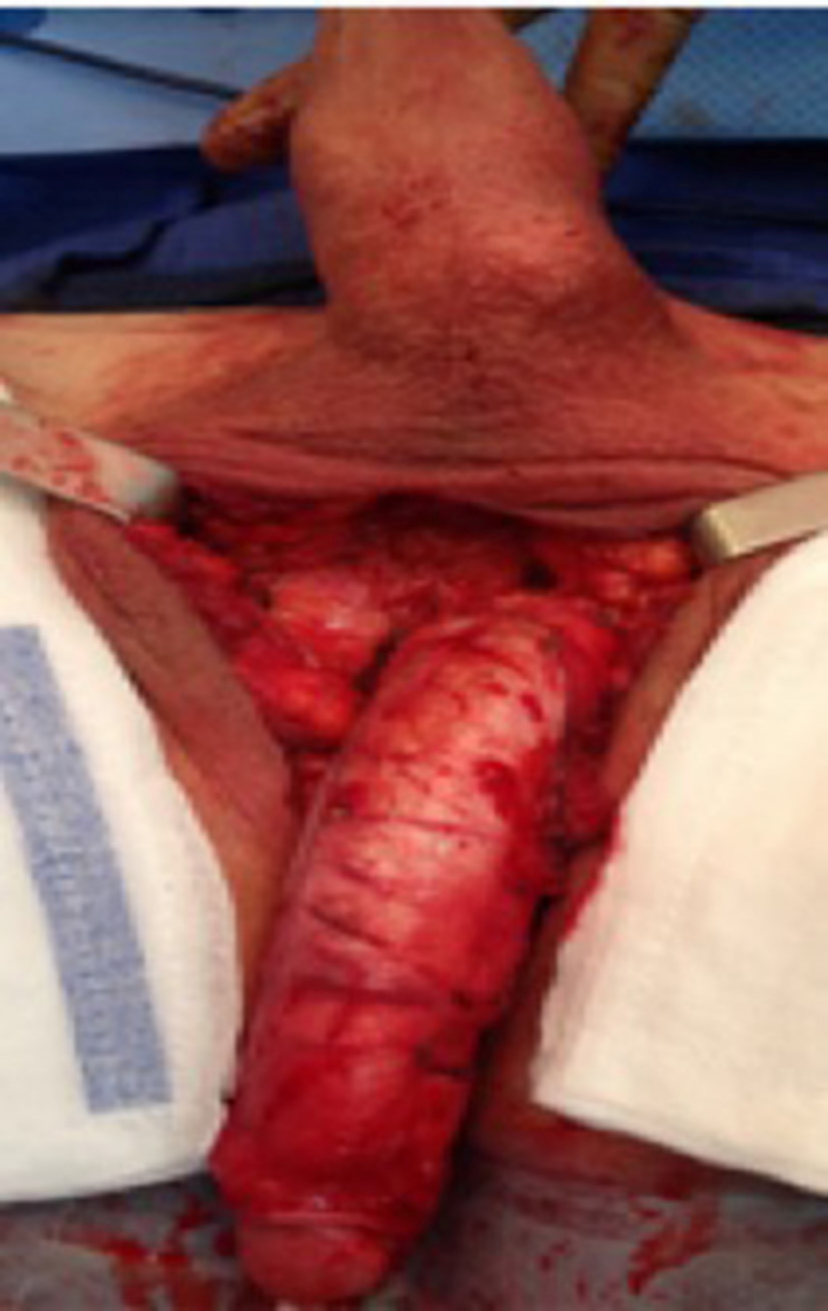
The de-gloved penis being passed through the scrotal opening.

**Figure 3:**
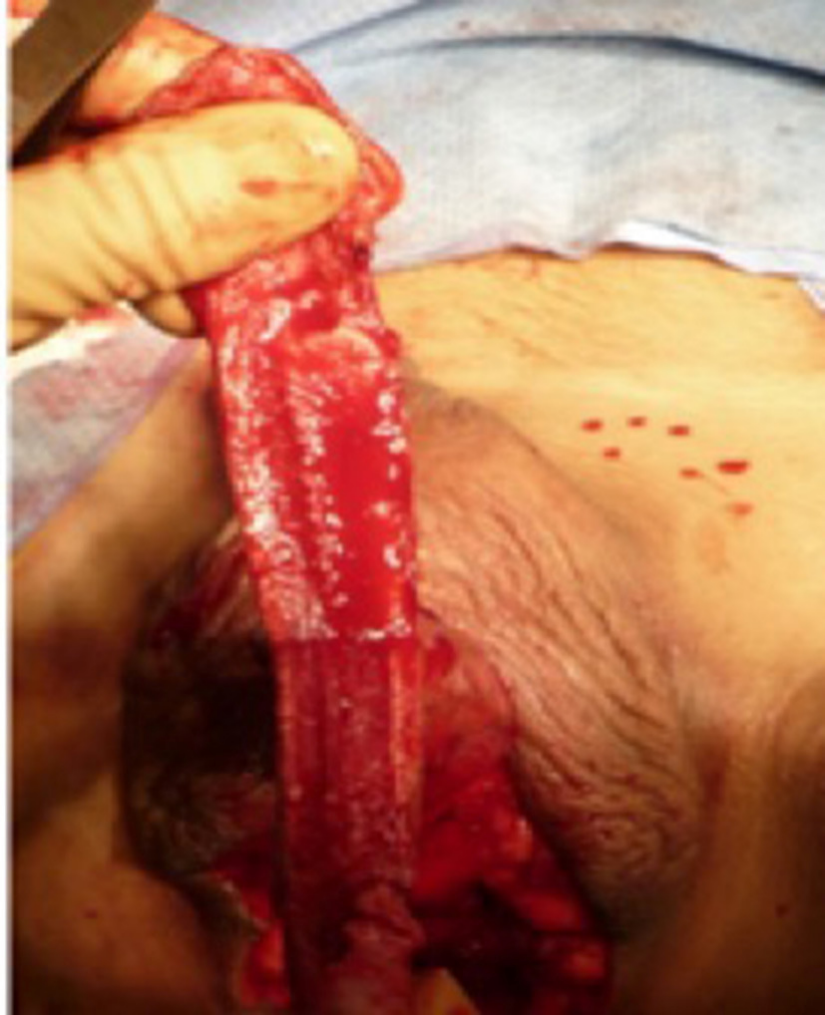
The dorsal part of the glans and its neurovascular bundle dissected away from the penile shaft.

**Figure 4:**
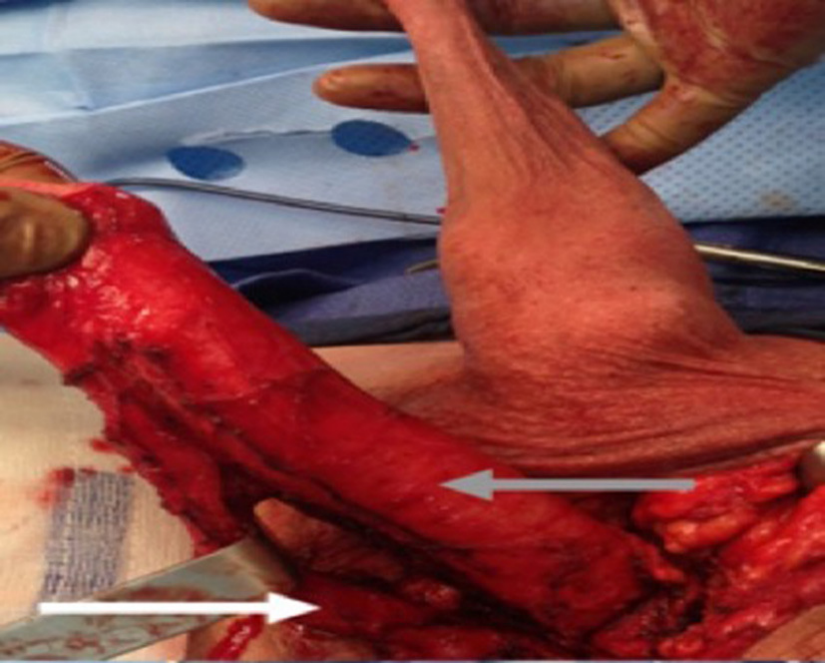
The urethra dissected including a portion of the bulbospongious muscle. The grey arrow shows the penile shaft and the white arrow shows the dissected urethra.

**Figure 5:**
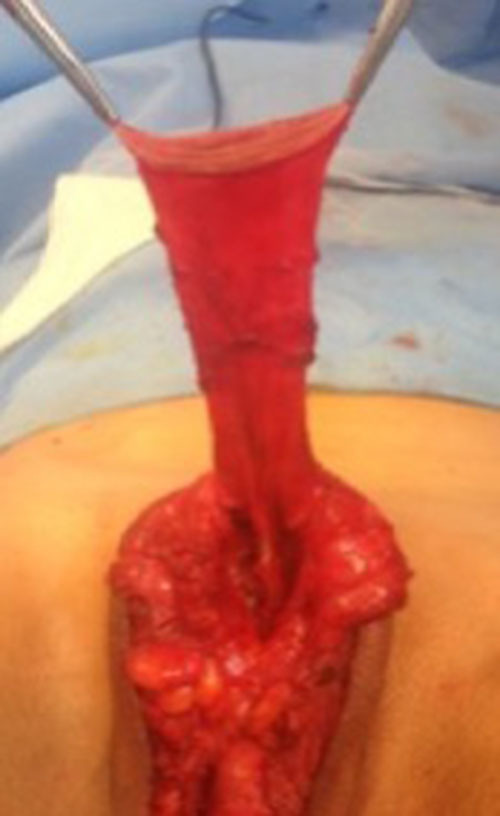
The inverted penile skin flap.

**Figure 6:**
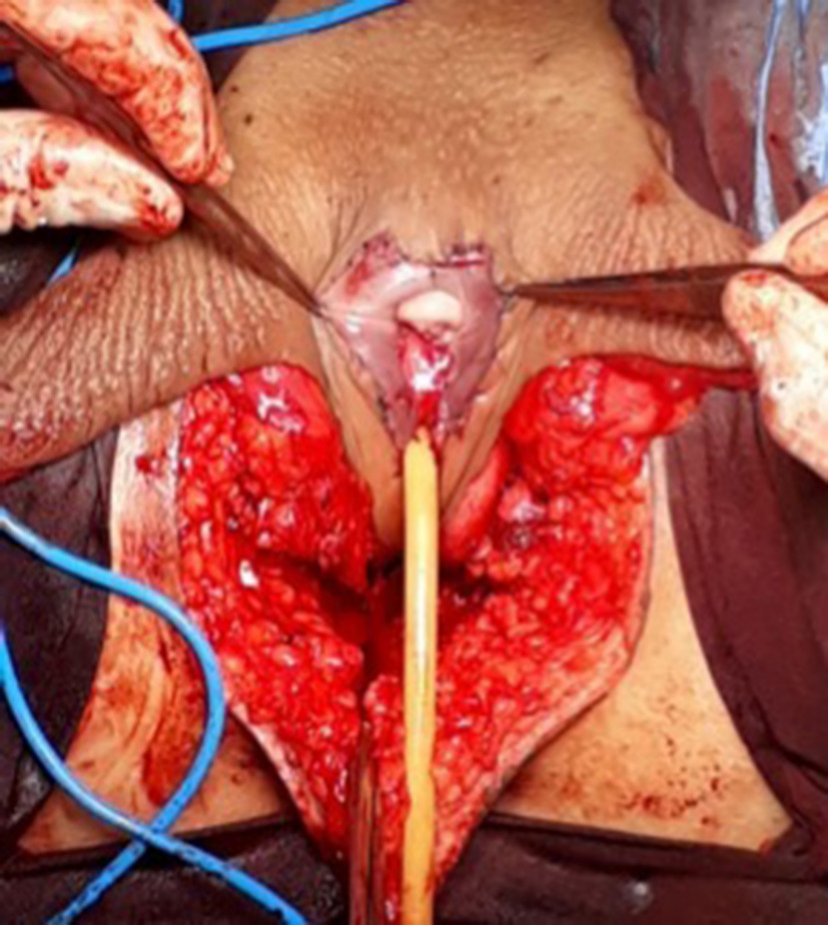
The neoclitoris and the urethra sutured in the midline and the neovaginal cavity.

**Figure 7:**
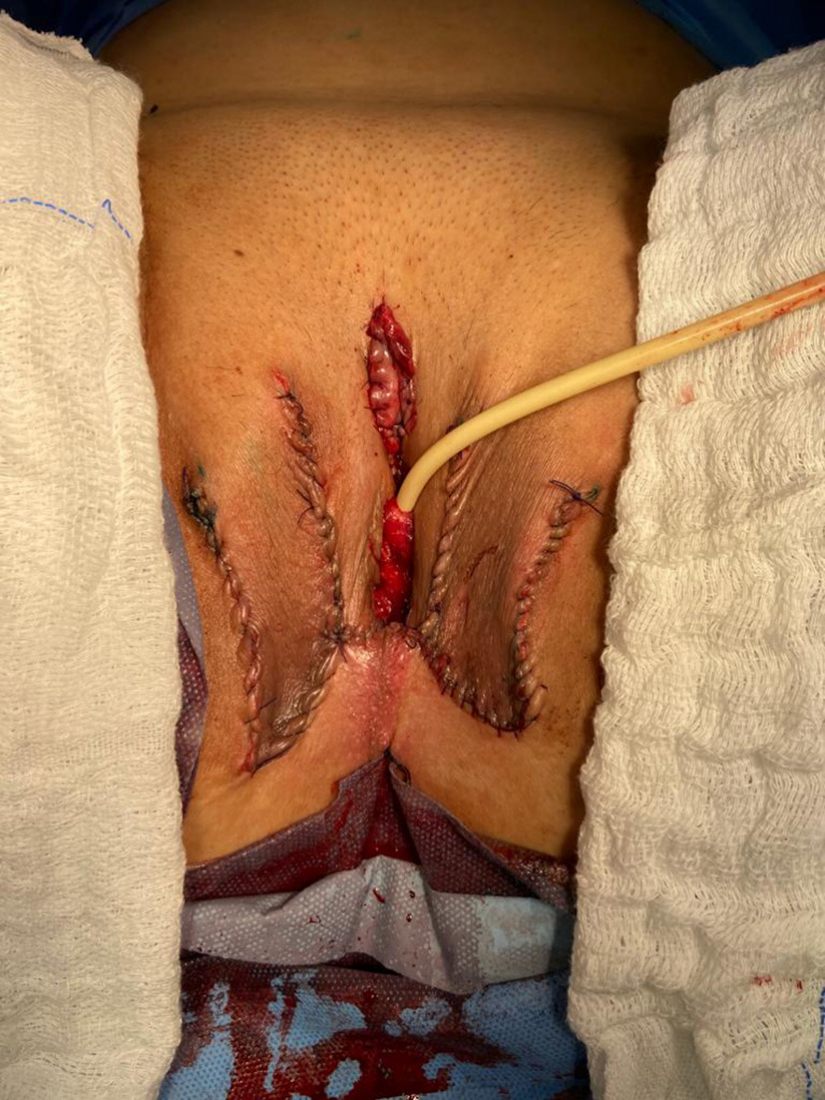
The final appearance after the completion of the procedures.

At our institution, we perform the classic penile inversion vaginoplasty, with an inverted penis skin flap used as the lining for the neovagina. For more details, we have previously published our technique with a step-by-step procedure video (6). All individuals underwent intestinal cleansing the evening before the surgery. A first-generation cephalosporin was used as preoperative prophylaxis. The procedure was performed with the patient in a dorsal lithotomy position. A Foley catheter was placed for bladder catheterization. A inverted-V incision was made 4 cm above the anus and a flap was created. A neovaginal cavity was created between the prostate and the rectum with blunt dissection, in the Denonvilliers space, until the peritoneal fold, usually measuring 12 cm in extension and 6 cm in width. The incision was then extended vertically to expose the testicles and the spermatic cords, which were removed at the level of the external inguinal rings. A circumferential subcoronal incision was made (Figure 1), the penis was de-gloved and a skin flap was created, with the de-gloved penis being passed through the scrotal opening (Figure 2). The dorsal part of the glans and its neurovascular bundle were bluntly dissected away from the penile shaft (Figure 3) as well as the urethra, which included a portion of the bulbospongious muscle (Figure 4). The corpora cavernosa was excised up to their attachments at the symphysis pubis and ligated. The neoclitoris was shaped and positioned in the midline at the level of the symphysis pubis and sutured using interrupted 5-0 absorbable suture. The corpus spongiosum was reduced and the urethra was shortened, spatulated, and placed 1 cm below the neoclitoris in the midline and sutured using interrupted 4-0 absorbable suture. The penile skin flap was inverted and pulled into the neovaginal cavity to become its walls (Figure 5). The excess of skin was then removed, and the subcutaneous tissue and the skin were closed using continuous 3-0 non-absorbable suture (Figure 6). A neo mons pubis was created using a 0 absorbable suture between the skin and the pubic bone. The skin flap was fixed to the pubic bone using a 0 absorbable suture. A gauze impregnated with Vaseline and antibiotic ointment was left inside the neovagina, and a customized compressive bandage was applied (Figure 7—shows the final appearance after the completion of the procedures).

### Postoperative Care and Follow-Up

The patients were usually discharged within 2 days after surgery with the Foley catheter and vaginal gauze packing in place, which were removed after 7 days in an ambulatorial attendance.

Our vaginal dilation protocol starts seven days after surgery: a kit of 6 silicone dilators with progressive diameter (1.1–4 cm) and length (6.5–14.5 cm) is used; dilation is done progressively from the smallest dilator; each size should be kept in place for 5 min until the largest possible size, which is kept for 3 h during the day and during the night (sleep), if possible. The process is performed daily for the first 3 months and continued until the patient has regular sexual intercourse.

**Figure 8:**
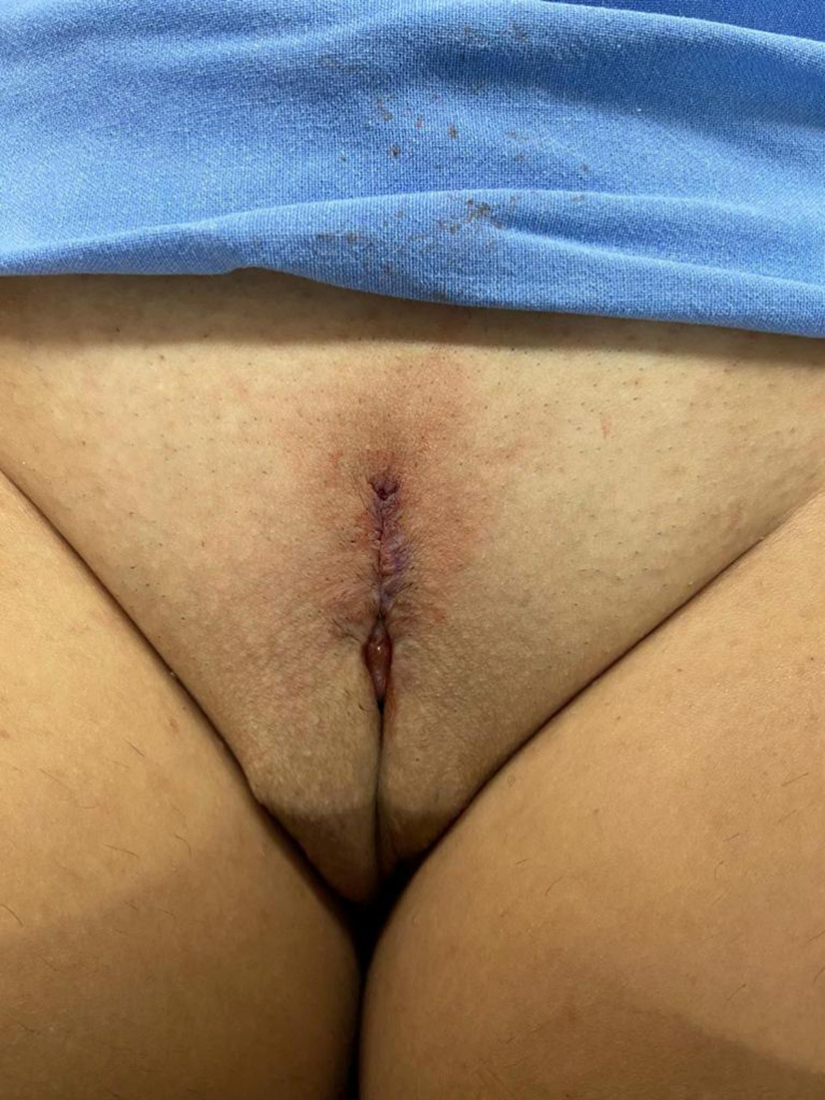
Appearance after 1 month of the procedure.

The follow-up visits were performed 7 days, 1, 2, 3, 6, and 12 months after surgery (Figure 8), and included physical examination and a quality-of-life questionnaire.

### Statistical Analysis

The statistical analysis was conducted using Statistical Product and Service Solutions Version 18.0 (SPSS). Outcome measures were intra-operative and postoperative complications, re-operations. Descriptive statistics were used to evaluate the study outcomes. Mean values and standard deviations or median values and ranges are presented as continuous variables. Frequencies and percentages are reported for dichotomous and ordinal variables.

## Results

### Patient Demographics

During the period of the study, 214 patients underwent penile inversion vaginoplasty, performed by two staff surgeons, mostly assisted by residents (Table 1). The average age at the time of surgery was 32.2 years (range 18–61 years). There was no significant increase or decrease in the ages of patients who underwent SRS over the study period (Fisher's exact test: *P* = 0.065; chi-square test: *X*^2^ = 5.15; GL = 6; *P* = 0.525). The average of operative time was 3.3 h (range 2–5 h). The average duration of hormone therapy before surgery was 12 years (range 1–39). The majority of patients were white (88.3 percent). The most prevalent patient comorbidities were history of tobacco use (15 percent), human immunodeficiency virus infection (13 percent) and hypertension (10.7 percent). Other comorbidities are listed in Table 1.

**Table 1:** Patient demographics.

	**No. (%)**
Total no. of patients	214
**Age, yr**	
Average	32.2
Range	18–61
**Ethnicity**	
White	189 (88.3)
Non-white	25 (11.7)
**Comorbidities**	
History of tobacco use	32 (15.0)
HIV	28 (13.0)
Hypertension	23 (10.7)
Diabetes	14 (6.5)
Pulmonary disease	14 (6.5)
History of alcohol use	14 (6.5)
Liver disease/hepatitis	9 (4.2)
Length of time on hormones, yr^*^	
Average	12
Range	1–39
**Operative time, hr**	
Average	3.3
Range	2–5

*HIV, human immunodeficiency virus*.

* *214 patients with available data*.

Multidisciplinary follow-up was comprised of 93.45% of patients following up with a urologist and 59.06% of patients continuing psychiatric follow-up, median follow-up time of 16 and 9.3 months after surgery, respectively.

### Postoperative Results

The complications were classified according to the Clavien-Dindo score (Table 2). The most common minor postoperative complications (Grade I) were granulation tissue (20.5 percent), introital stricture of the neovagina (15.4 percent) and wound dehiscence (12.6 percent). The major complications (Grade III-IV) included urethral stenosis (20.5 percent), urethral fistula (1.9 percent), intraoperative rectal injury (1.9 percent), necrosis (primarily along the wound edges) (1.4 percent), and rectovaginal fistula (0.9 percent). A total of 17 patients required blood transfusion (7.9 percent).

**Table 2:** Complications after penile inversion vaginoplasty.

	**No. (%)**
Total no. of patients	214
Patients with any complications	82 (44%)
**Grade 1 complications**	
Granulation tissue	44 (20.5)
Introital stricture	33 (15.4)
Wound dehiscence	27 (12.6)
Hematoma/excessive bleeding	19 (8.9)
Tissue necrosis	4 (1.9)
**Grade 2 complications**	
Need for transfusion	17 (7.9)
**Grade 3 or 4 complications**	
Urethral meatus strictures	44 (20.5)
Urethral fistula	4 (1.9)
Intraoperative rectal injury	4 (1.9)
Rectovaginal fistula	2 (0.9)

A total of 36 patients (16.8 percent) underwent some form of reoperation.

One hundred eighty-one (85 percent) patients in our series were able to have regular sexual vaginal intercourse, and no individual regretted having undergone GAS.

## Discussion

Penile inversion vaginoplasty is the gold-standard in gender-affirming surgery. It has good functional outcomes, and studies have demonstrated adequate vaginal depths (3). It is recognized not only as a cosmetic procedure, but as a therapeutic intervention and a medical necessity (2). We present the largest South-American cohort to date, examining demographic data, intra and postoperative complications.

The mean age of transsexual women who underwent GAS in our study was 32.2 years (range 18–61 years), which is lower than the mean age of patients in studies found in the literature. Two studies indicated that the mean ages of patients at time of GAS were 36.7 years and 41 years, respectively (4, 5). Another study reported a mean age at time of GAS of 36 years and found there was a significant decrease in age at the time of GAS from 41 years in 1994 to 35 years in 2015 (7). According to the authors, this decrease in age is associated with greater tolerance and societal approval regarding individuals with GD (7).

There was no grade IV or grade V complications. Excessive bleeding noticed postoperatively occurred in 19 patients (8.9 percent) and blood transfusion was required in 17 cases (7.9 percent); all patients who required blood transfusions were operated until July 2011, and the reason for this rate of blood transfusion was not identified.

The most common intraoperative complication was rectal injury, occurring in 4 patients (1.9 percent); in all patients the lesion was promptly identified and corrected in 2 layers absorbable sutures. In 2 of these patients, a rectovaginal fistula became evident, requiring fistulectomy and colonic transit deviation. This is consistent with current literature, in which rectal injury is reported in 0.4–4.5 percent of patients (4, 5, 8–13). Goddard et al. suggested carefully checking for enterotomy after prostate and bladder mobilization by digital rectal examination (4). Gaither et al. (14) commented that careful dissection that closely follows the urethra along its track from the central tendon of the perineum up through the lower pole of the prostate is critical and only blunt dissection is encouraged after Denonvilliers' fascia is reached. Alternatively, a robotic-assisted approach to penile inversion vaginoplasty may aid in minimizing these complications. The proposed advantages of a robotic-assisted vaginoplasty include safer dissection to minimize the risk of rectal injury and better proximal vaginal fixation. Dy et al. (15) has had no rectal injuries or fistulae to date in his series of 15 patients, with a mean follow-up of 12 months.

In our series, we observed 44 cases (20.5 percent) of urethral meatus strictures. We credit this complication to the technique used in the initial 5 years of our experience, in which the urethra was shortened and sutured in a circular fashion without spatulation. All cases were treated with meatal dilatation and 11 patients required surgical correction, being performed a Y-V plastic reconstruction of the urethral meatus. In the literature, meatal strictures are relatively rare in male-to-female (MtF) GAS due to the spatulation of the urethra and a simple anastomosis to the external genitalia. Recent systematic reviews show an incidence of five percent in this complication (16, 17). Other studies report a wide incidence of meatal stenosis ranging from 1.1 to 39.8 percent (4, 8, 11).

Neovagina introital stricture was observed in 33 patients (15.4 percent) in our study and impedes the possibility of neovaginal penetration and/or adversely affects sexual life quality. In the literature, the reported incidence of introital stenosis range from 6.7 to 14.5 percent (4, 5, 8, 9, 11–13). According to Hadj-Moussa et al. (18) a regimen of postoperative prophylactic dilation is crucial to minimize the development of this outcome. At our institution, our protocol for vaginal dilation started seven days after surgery and was performed three to four times a day during the first 3 months and was continued until the individual had regular sexual intercourse. We treated stenosis initially with dilation. In case of no response, we propose a surgical revision with diamond-shaped introitoplasty with relaxing incisions. In recalcitrant cases, we proposed to the patient a secondary vaginoplasty using a full-thickness skin graft of the lower abdomen.

One hundred eighty-one (85 percent) patients were classified as having a “functional vagina,” characterized as the capacity to maintain satisfactory sexual vaginal intercourse, since the mean neovaginal depth was not measured. In a review article, the mean neovaginal depth ranged from 10 to 13.5 cm, with the shallowest neovagina depth at 2.5 cm and the deepest at 18 cm (17). According to Salim et al. (19), in terms of postoperative functional outcomes after penile inversion vaginoplasty, a mean percentage of 75 percent (range from 33 to 87 percent) patients were having vaginal intercourse. Hess et al. found that 91.4% of patients who responded to a questionnaire were very satisfied (34.4%), satisfied (37.6%), or mostly satisfied (19.4%) with their sexual function after penile inversion vaginoplasty (20).

Poor cosmetic appearance of the vulva is common. Amend et al. reported that the most common reason for reoperation was cosmetic correction in the form of mons pubis and mucosa reduction in 50% of patients (16). We had no patient regrets about performing GAS, although 36 patients (16.8 percent) were reoperated due to cosmetic issues. Gaither et al. propose in order to minimize scarring to use a one-stage surgical approach and the lateralization of surgical scars to the groin (14). Frequently, cosmetic issues outcomes are often patient driven and preoperative patient education is necessary (14).

Analyzing the quality of life, in 2016, our health care group (PROTIG) published a study assessing quality of life before and after gender-affirming surgery in 47 patients using the diagnostic tool 100-item WHO Quality of Life Assessment (WHOQOL-100) (21). The authors found that GAS promotes the improvement of psychological aspects and social relations. However, even 1 year after GAS, MtF persons continue to report problems in physical and difficulty in recovering their independence. In a systematic review and meta-analysis of QOL and psychosocial outcomes in transsexual people, researchers verified that sex reassignment with hormonal interventions more likely corrects gender dysphoria, psychological functioning and comorbidities, sexual function, and overall QOL compared with sex reassignment without hormonal interventions, although there is a low level of evidence for this (22). Recently, Castellano et al. assessed QOL in 60 Italian transsexuals (46 transwomen and 14 transmen) at least 2 years after SRS using the WHOQOL-100 (general QOL score and quality of sexual life and quality of body image scores) to focus on the effects of hormonal therapy. Overall satisfaction improved after SRS, and QOL was similar to the controls (23). Bartolucci et al. evaluated the perception of quality of sexual life using four questions evaluating the sexual facet in individuals with gender dysphoria before SRS and the possible factors associated with this perception. The study showed that approximately half the subjects with gender dysphoria perceived their sexual life as “poor/dissatisfied” or “very poor/very dissatisfied” before SRS (24).

Our study has some limitations. The total number of operated patients is restricted within the long follow-up period. This is due to a limitation in our health system, which allows only 1 sexual reassignment surgery to be performed per month at our institution. Neovagin depth measurement was not performed routinely in the follow-up of operated patients.

## Conclusions

The definitive treatment for patients with gender dysphoria is gender-affirming surgery. Our series demonstrates that GAS is a feasible surgery with low rates of serious complications. We emphasize the high level of functionality of the vagina after the procedure, as well as subjective personal satisfaction. Complications, especially minor ones, are probably underestimated due to the nature of the study, and since this is a surgical population, the results may not be generalizable for all transgender MTF individuals.

## Data Availability Statement

The raw data supporting the conclusions of this article will be made available by the authors, without undue reservation.

## Ethics Statement

The studies involving human participants were reviewed and approved by Hospital de Clínicas de Porto Alegre. The patients/participants provided their written informed consent to participate in this study.

## Author Contributions

GM: conception and design, data acquisition, data analysis, interpretation, drafting the manuscript, review of the literature, critical revision of the manuscript and factual content, and statistical analysis. ML and TR: conception and design, data interpretation, drafting the manuscript, critical revision of the manuscript and factual content, and statistical analysis. DS, KS, AF, AC, PT, AG, and RC: conception and design, data acquisition and data analysis, interpretation, drafting the manuscript, and review of the literature. All authors contributed to the article and approved the submitted version.

## Conflict of Interest

The authors declare that the research was conducted in the absence of any commercial or financial relationships that could be construed as a potential conflict of interest.
